# Endowments

**Published:** 1894-01-27

**Authors:** 


### THE INSTITUTIONAL WORKSHOP.

### HOSPITAL FINANCE.

### II.—ENDOWMENTS.

From very early times in the existence of hospitals, endowments have proved the most popular of all sources of income. In days when but a very small minority were at all concerned about the sufferings of the sick-poor, hospital work could not, indeed, have been even attempted without them. Rich gifts and bequests were freely forthcoming, for to almost every mind there is an attraction in assigning a large sum to work out a definite object for untold generations to come; there is unconsciously a sense of the testator's superiority in wisdom and benevolence to his descendants yet unborn, and a pathetic trust in the power of his money to work out after his death the good he has bent his energies to achieving during his life-time. For experience shows that it is the steady, continuous benefactor of the hospital who is mindful of its claims in his will. That the endowments should be eagerly welcomed by the hospitals needs no explanation. Money acquired without effort, and for the most part unhampered by restrictions, must always appear in the aspect of an unmixed good. Yet, viewed in the light of history, the endowment system is seen to be one long record of miserable failure. Many centuries of experience have proved that money left in the hands of close corporations has resulted in educating those charged with its administration in a habit of singular irresponsibility, leading almost inevitably, by the path of reckless wastefulness, to direct misappropriation of the funds. The proportion of the income swallowed up in official salaries tends to an excessive increase. The actual capital has been not infrequently wrecked for want of the most ordinary business diligence, while the purpose of the founder being completely thrust on one side, his true legatees find themselves insolently treated as paupers, and deprived of the greater part of what is unquestionably their lawful patrimony. It would seem that the very stars in their courses have fought against the endowed charity. The needs of one generation have been outgrown in the next, and the laws framed to protect the trust from spoliation serve only as a fatal barrier to its adaptation to altered conditions. Perhaps the saddest part of the whole system has been that abuses crying aloud for redress have flourished, not only under the fraudulent and self-interested, but under the fostering care of the upright, the pious, and the benevolent, whose standard of morality has been insensibly corrupted to acquiescence in evils from which they have noted each other to be not ashamed to profit. It is clear that the administration of the money of the dead is fraught with so many and subtle temptations, that every safeguard which entire publicity and skilled scrutiny can furnish, should be welcomed by those on whom the heavy burden falls. It will be useful to examine briefly the results of endowment on hospital efficiency.

In few countries has a more liberal provision been set aside by successive generations for the benefit of their poorer posterity, than in Italy. The result of investigations set on foot by Signor Crispi, in 1890, revealed that the property of the Opere Pie, or pious foundations, amounted to a total sum of £80,000,000 with a yearly income of £3,600,000, capable by better management of being increased to £4,000,000. Yet out of this vast sum, but £400,000 was available for the support of strictly charitable institutions. In some of these establishments it was found that the cost of administering the charity was more than a third part of the whole income. In general there was the complaint that the priests managed them either for their own comfort or for political purposes. It was said for instance that in Palermo of the large funds which come into the hands of the clerical managers of these charities, only a small part goes to the poor, the rest being used politically, and as was intimated, against the existing Government of Italy. With respect to hospitals Italy is far behind other continental nations. From all its hospitals contagious diseases are rigidly excluded, yet the original statutes of these institutions prove that in numerous cases the wealthy endowments were left exclusively for the treatment of persons suffering from leprosy and other infectious disorders imported from the East. The condition of the richly-endowed hospital of San Giacomo in Rome may be taken as a specimen of the result of irresponsible management of inalienable funds. "I saw the so-called camerette (closets) in which the cancer patients were confined. They are low-roofed, without air, without light, 'indecentissimi.' But what roused my indignation most were the buchi (holes) where some chronic female patients were accommodated. One of these has been an inmate of the San Giacomo since 1848, another since 1855. These unfortunate creatures are kept under a staircase. Considering that they refused to die, and that they were incurable, they were stowed away like superannuated furniture." This hospital has since been placed under a Royal Commissioner. All the charities in Italy were reorganised in 1890, and brought under the direction of boards elected in each commune, and these charitable funds are now largely supplemented by taxation. It would be grossly unfair to attribute the mismanagement simply to clerical influence.

In France the charitable endowments, especially for hospital work, are very considerable, and date back for many centuries. The usual story of mismanagement and misappropriation of funds follows each institution, and unanimously the citizens made demand of the Deputies sent up to the National Assembly of 1789 that radical changes should be effected in the hospital system. As a matter of fact, the French have rearranged their charitable systems so often since that date that it is no easy matter to ascertain what proportion the old endowments bear towards the cost of fulfilling the duty now undertaken by the State of caring for the sick poor. The endowments had a narrow escape of perishing altogether in the new fervour of the Revolution by a law passed July 11th, 1794, assimilating hospital property with the national wealth, and rendering it liable to alienation, the maintenance of the sick poor devolving on the State. The following year this sale of hospital property was, however, first deferred, and then put a stop to altogether. Each institution has now its own endowments secured to it, under the strictest supervision and State control. These funds are supplemented, according to the character of the institution, by voluntary offerings and by communal grants.

Owing to the fact that in Germany the control of most of the hospitals fell under the direction of the towns, and that they were open therefore to public criticism, the funds have been on the whole satisfactorily managed. Many endowments it is true fell a prey to the warlike nobles or were diverted to other purposes, but hospital work has been steadily supported from very early times, and many of the oldest foundations are still in existence and doing good work without having undergone the ordeal of any violent reorganisation. The endowments are largely supplemented by voluntary offerings.

In England the endowed, as distinguished from the voluntary, hospitals are three in number; St. Bartholomew's and St. Thomas's (known as the Royal Hospitals), and Guy's Hospital, Southwark. The combined incomes of the three averages about £150,000 a year, and they have accommodation for about 1,600 patients. It is needless to recall the adverse criticisms which have been freely directed against these institutions from time to time since their foundation. It is probable that these very criticisms, often ignorant or even malicious, have had their part in preserving the English hospitals from the more miserable wreckage which has sometimes befallen, as we have seen, similar institutions abroad. It has not preserved them from many grave defects. At the present day the administration of the three endowed hospitals is undoubtedly good, though in one or two notorious points it might easily be better. Yet out of the three St. Bartholomew's only can be said to be in a financially strong position. "Mistakes on the part of a late treasurer and governor of St. Thomas's brought about disastrous results so far as its finances were concerned, and for many years it has been crippled in consequence in its work. Guy's Hospital is even in a worse plight, because there the absence of an active interest in the administration on the part of the governing body has resulted in the missing of opportunities to fully develop the estates and to anticipate changes in the value of agricultural property which might easily have been foreseen by the exercise of common prudence." And it is hardly sufficient that the endowed hospitals should barely attain the standard reached by institutions which are obliged to maintain yearly a desperate struggle for existence. It might reasonably be expected that they should lead the way in wise liberality to those who spend their lives in devoted attendance on the sick within their walls, and in inaugurating the best methods of healing which modern science can suggest. Perhaps nothing more forcibly illustrates the wisdom of trusting each generation to perform its own work than a comparison of the successive stages of progress achieved in the voluntary and endowed hospitals respectively. And the conclusion is irresistible that no greater misfortune can befall a hospital than an endowment fund so large as to preclude the necessity for popular support.

It must be added that in the majority of voluntary hospitals the anxiety of living entirely from hand to mouth has brought so heavy a strain upon the managers that a certain proportion of donations and legacies has been set aside—say a sum equal to three years' expenditure as a maximum, if they are wise—to provide a small endowment fund, and meet any sudden emergency.

